# Testing the Attractive Appeal of *Desmodium* Infochemicals to Key Parasitoids of the Vegetable Integrated Push–Pull Cropping System

**DOI:** 10.1007/s10886-025-01622-1

**Published:** 2025-07-04

**Authors:** Frank Chidawanyika, Bretor K. Mutua, Isack H. Adan, Daniel M. Mutyambai

**Affiliations:** 1 International Centre of Insect Physiology and Ecology (Icipe), Nairobi, Kenya; 2 Department of Zoology and Entomology, University of the Free State, P.O. Box 339, Bloemfontein, 9300 South Africa; 3 Department of Life Sciences, South Eastern Kenya University, P.O Box 170-90200, Kitui, Kenya

**Keywords:** Biological control, Habitat management, Host selection, Indirect defence, Plant volatiles, Trophic interactions

## Abstract

Biological control agents heavily rely on volatile cues for host location and can be an important component of managing pests through habitat management-based strategies that enhance trophic interactions. This study aimed at evaluating the influence of greenleaf desmodium (*Desmodium intortum* (Mill.) Urb. (Fabaceae) infochemicals on the behaviour of three select parasitoids of kale pests (aphids and Diamondback moth), to determine the potential of enhancing the protection of vegetables in integrated push–pull cropping systems. To achieve this, the volatile-mediated behavioural responses of the parasitoids towards *D*. *intortum* volatiles were evaluated using a dual-choice Y-tube olfactometer. Our results showed that *Cotesia vestalis* (Hymenoptera: Braconidae), a parasitic wasp for Diamondback moth (DBM) (*Plutella xylostella* (Linneaus)) was not attracted to volatiles from *D. intortum* compared to DCM and empty oven bag controls. Nevertheless, *D*. *intortum* VOCs elicited higher parasitoid activity, whether in dual-choice comparisons with either empty oven bags or DCM controls, or when paired with kale plants. Conversely, volatiles from D. *intortum* were highly attractive to *Aphidius* colemani (Viereck) (Hymenoptera: Braconidae), a parasitic wasp of aphids. Interestingly, another aphid parasitic wasp, *Aphidius ervi* (Haliday) (Hymenoptera: Braconidae) was attracted to volatiles from *D. intortum* only when paired with kales or by kales alone. Gas-chromatography mass spectrometry of *D. intortum* headspace volatiles revealed 16 compounds: hexanal, (*Z*)-3-hexenol, *p*-xylene, *o*-xylene, nonane, α-pinene, cumene, octen-3-ol, octanone, (*Z*)-3-hexenyl acetate, sabinene, (*E*)-β-ocimene, linalool, β-elemene, (*E*)-β-caryophyllene and an unknown compound. In electroantennography assays, all parasitoid antennae commonly detected hexanal and, (*E*)-β-ocimene, whilst only *C. vestalis* and *A. ervi* registered common antennal responses to (*E*)-β-caryophyllene. Additionally, the antennae of *A*. *colemani* detected cumene, octen-3-ol, (*Z*)-3-hexenyl acetate and an unknown compound whilst that of *A. ervi* and *C*. *vestalis* detected (*Z*)-3-hexenol and nonane, respectively. Dose–response olfactometer bioassays with the synthetic standards of hexanal, (*E*)-β-ocimene and (*E*)-β-caryophyllene revealed that the response of the three parasitic wasps varied with the concentrations of the individual standards. Specifically, (*E*)-β-ocimene and hexanal depicted a broad appeal to the tested parasitoids, by eliciting attraction at varying concentrations (*P* < 0.05). However, (*E*)-β-caryophyllene was selectively attractive to *A*. *ervi*, with no significant attraction observed in *C*. *vestalis* (*P* > 0.05). Our results show species- and -context -specific parasitoid attractive appeal of *D. intortum*. Nevertheless, our results show that *D. intortum* may help protect kales against aphid attack through parasitoid recruitment whilst another mechanism may be employed against DBM moth.

**Supplementary Information:**

The online version contains supplementary material available at 10.1007/s10886-025-01622-1.

## Introduction

Companion cropping can boost crop productivity through various ecosystem services, below and above ground, through improved soil health, crop protection and pollination services (Khan et al. 2014; Chidawanyika et al. 2023). For crop protection, aromatic companion plants can mediate trophic interactions influencing chemical profiles that can repel crop pests or attract their natural enemies (Mumm and Dicke 2010; Odermatt et al. 2024). Floral resources of natural enemies can also aid in the conservation of natural enemies by providing food (Parolin et al. 2012). Such companion cropping has proven successful in curbing various pests including cabbage root fly and onion fly (Finch et al. 2003), cereal stemborers and fall armyworm (Cheruiyot et al. 2021a, b; Sobhy et al. 2022), cruciferous vegetable pests (Chidawanyika et al. 2023, 2025; Mutua et al. 2024), millet shoot fly, (Moreno and Racelis 2015) and cotton pests (Midega et al. 2012; Chi et al. 2021). The soil legacies of such companion cropping can indirectly mediate arthropod assemblages through changes in crop secondary metabolites that can minimize crop damage (Lang et al. 2024; Mutyambai et al. 2019, 2023).

One of the most successful forms of companion cropping, the push–pull or ‘stimulo-deterrent diversionary’ strategies, which integrate various cues to manipulate pest behaviour by both repulsion and attraction away from the crop and has been tested in various systems since seminal work on various crops (Kennedy 1965; Dethier 1982; Prokopy and Owens 1983; Miller and Strickler 1984). More recently, the strategy has been employed in managing cereal crop pests [*Busseola fusca* (Fuller), *Chilo partellus* (Swinhoe)) and fall armyworm (*Spodoptera frugiperda* (JE Smith)] in Africa to form what is now commonly referred to as push–pull technology (PPT). This PPT relies on combined attractive and deterrent cues from perennial border crops, mainly *Desmodium* spp. (push) and *Brachiaria brizantha* (pull), respectively (Khan et al. 2014). Typically, *Desmodium* spp. emit homoterpenes, monoterpenes and sesquiterpenes, such as (*E*)−4,8-dimethyl-1,3,7-nonatriene ((*E*)-DMNT) and (*E*)-β-ocimene and (*E*)-β-caryophyllene, that are responsible for insect deterrence and parasitoid recruitment (Khan et al. 2014; Sobhy et al. 2022). *Desmodium* spp. also enhance soil microbial activity and inhibits the noxious weed (*Striga* spp.) post-germination by inducing an allelopathic suicidal germination mechanism (Tsanuo et al. 2003). Additionally, it improves soil organic matter, phosphorus bioavailability, and soil nitrogen via biological nitrogen fixation (BNF) (Ndayisaba et al. 2022). On the other hand, the pull plants act as ecological traps by emitting VOC attractants that lure pests away from the main crop (Khan and Pickett 2004) but arrest development using the gummy substance produced in their stems due to the feeding stemborer larvae ultimately leading to larval mortality (Khan et al. 2014).

Nearly three decades since its inception, the PPT cropping system has evolved with tailored adjustments to enhance its resilience against global climate change and other biophysical constraints (Chidawanyika et al. 2014; Cheruiyot et al. 2021a, 2021b). More recently, high-value vegetables crops, have since been incorporated in PPT, forming the vegetable integrated push–pull system (VIPP) in a bid to improve both nutritional and socioeconomic outcomes (Chidawanyika et al. 2023). Interestingly, field evaluations have shown that most vegetables integrated into the VIPP substantially enjoy crop protection benefits with improved yield (Chidawanyika et al. 2023, 2025). The mechanistic basis of such improved protection remains poorly explored but is highly likely that the same *Desmodium* spp. infochemicals responsible for cereal protection in PPT (Khan et al. 1997; Sobhy et al. 2022) may influence vegetable-pest interactions and partly through enhanced parasitoid recruitment.

Parasitoids are a diverse group of insects that play a critical role in biological pest control of devastating pests via antagonistic interactions (Midega et al. 2009). Here, we evaluated three key parasitoids of vegetable pests namely *C*. *vestalis*, *A. ervi*, and *A. colemani*. *Cotesia vestalis* is a solitary larval parasitoid known for its role in combatting DBM, a destructive pest of cruciferous vegetables worldwide (Saini et al. 2019). The DBM has been proven to cause marketable yield loses ranging from 50 to 90%, depending on the crop and infestation levels (Oke 2008). The DBM is also very adept at developing resistance to insecticides (Saini et al. 2019), making the demand for biological control and alternative integrated pest management strategies an urgent requirement. The cosmopolitan endoparasitoids, *A*. *ervi and A. colemani,* target a number of aphid species such as green peach aphid (*Myzus persicae* (Sulzer)), the cotton aphid (*Aphis gossypii*), tobacco aphid (*Myzus persicae* subsp*.* nicotianae) and cabbage aphids (*Brevicoryne brassicae*) (Boivin et al. 2012).These targeted pests cause significant damage to crops either directly through feeding or indirectly, via disease transmission (Mutua et al. 2024). Previous studies on these parasitoids have shown that gravid parasitoids utilize VOCs blends as cues to locate their hosts for parasitisation (Ahmed et al. 2022; Girling et al. 2011; Uefune et al. 2020). Since *D*. *intortum* has shown success in attracting cereal-pest parasitoids, it would be imperative to evaluate its potential in attracting parasitoids in a VIPP system.

The current study evaluated the potential of *D*. *intortum* infochemicals in attracting *C. vestalis*, *A. ervi*, and *A. colemani* parasitoids. We hypothesized that the infochemicals attract these parasitoids, assisting them in locating their target host in a VIPP system thereby improving the protection of vegetables against pest damage.

## Materials and Methods

### Plants

Seeds of *D*. *intortum* and kale, *Brassica oleracea* var acephala (thousand-headed variety) were obtained from Simlaw Seeds Company Ltd (Nairobi, Kenya). *Brassica oleracea* seeds were first sterilized by soaking in 1% sodium hypochlorite solution to eliminate seed-borne pathogens then thoroughly rinsed in three changes of distilled water before drying using adsorbent paper. The sterile seeds were then sown in individual 5L plastic pots filled with autoclaved soil and manure in the ratio 2:1 (autoclaved for 30 min at 121℃). *Desmodium intortum* seeds were surface sterilized as described above, rinsed, then pre-germinated in Petri dishes containing wet filter papers at room temperature. After radicle emergence, three seedlings were transplanted to 5L pots containing autoclaved soil without fertilizer. The plants were later thinned to one plant per pot after 21 days to reduce competition. The plants were kept in an insect-proof screenhouse, maintained at 25 ± 2 °C, 60–70% relative humidity (RH) and L12:D12 light/dark photoperiod at the International Centre of Insect Physiology and Ecology (*icipe*), Duduville Campus, Nairobi, Kenya, (01º 13′ 25.6″ S 036º 53′ 49.1″ E, 1616 m.a.s.l). The seedlings were watered daily, and they did not receive any chemical treatment. Four weeks old *Brassica oleracea* and 8 weeks old *D. intortum* were used for the experiments.

### Insects

Larvae of DBM were collected from smallholder farms in Homabay County, Kenya. Potted *B. oleracea* plants were provided for mass-rearing of the obtained larvae. *Cotesia vestalis* cocoons were also collected from the smallholder farms and their progeny subsequently reared on second and third instar DBM larvae as their host. The DBM larvae were monitored for cocoon formation, then separated into different cages and used for bioassays after successful eclosion. Mummified aphids were also collected from farmer fields and kept in rearing cages. After emergence, adult wasps of *A. ervi* and *A. colemani* were kept in separate cages containing *B. brassicae* feeding on *B. oleracea*. Afterwards, the mummified aphids were further separated into different cages and used for bioassays after emergence. In all the experiments, DBM*and B. Brassicae* were kept in 50 × 80 × 40 Perspex cages, while the emerging wasps were separated in 40 cm × 50 cm × 40 cm well-ventilated Perspex rearing cages. The rearing environment was maintained at 28 ± 2 °C, 55 ± 5% RH. Newly emerged adult wasps were fed with 20% honey-water solution soaked in cotton balls. One-day-old gravid female wasps were used for bioassays.

### Behavioral Responses of *C. vestalis*, *A. ervi* and *A. colemani* using Y-tube Olfactometer

Behavioural responses of *C. vestalis*, *A. ervi* and *A. colemani* to headspace *D. intortum* VOCs were carried to determine the orientation of the adult wasps (Supplementary Table 1). The assays were carried out in a Y-tube olfactometer (arm = 10 cm, stem = 14 cm, diameter = 3 cm) held at an inclining position of 45℃ between the Y-tube and the horizontal plane. The olfactometer was placed on a black background, illuminated using a 20 V white, fluorescent tube from above in a dark room to avoid any visual distractions. For bioassays involving VOCs of *D. intortum*, one arm of the olfactometer held 10 µl aliquots of the *D. intortum* VOCs while the other arm held 10 µl of dichloromethane (DCM) as a control, in a two-way choice test. The samples were applied on filter paper (4 × 25 mm) using a micropipette (Drummond Scientific, Broomall, USA), which were then placed in the two glass arms attached at the top of the Y-tube olfactometer. Thereafter, we conducted bioassays using live plants, individually enclosed in heat-sterilised (100 ℃ for 12 h) polyethylene terephthalate (PET) bags (50 cm × 60 cm; Lifetime Brands Europe Ltd, Valepits Road, Birmingham). Enclosed live *D. intortum* and *B. oleracea* plants and their combinations were tied on each side of the olfactometer against one another or clean air bag controls (Supplementary Table 1). To minimize contamination of headspace plant VOCs with those from the soil, the whole pot containing the soil was covered with aluminum foil to the base of the plant, leaving only the aerial parts of the plant exposed. An electric-powered air-free vacuum pump (Model: DAA-V174-EB, GAST Manufacturing Company, Benton Harbor, Michigan, United States) was used to suck charcoal-filtered air into the Y-tube at a flow rate of 700 mL/min thereby creating a net flow rate of 350 mL/min into the arms. To eliminate positional bias, treatment odor sources were alternated between the arms after every replication. All insects were used only once to avoid confounding results due to acquired experience using clean olfactometers. Plants were replaced with fresh ones after testing 5 insects. An insect was considered to have made a choice if it moved at least 5 cm into any arm and stayed for more than 1 min over a 10 min observation period. In all cases, experiments were carried out in a controlled laboratory environment as described by (Adams et al. 2023), between 12:30 p.m. and 5:00 p.m. The parasitoids and test plants were moved to the bioassay room one hour prior to conducting the bioassays to allow them to acclimatize. In all tested combinations, 30 gravid female wasps without oviposition exposure were tested.

### Collection and Analysis of Volatiles

Headspace sampling technique was used to collect VOCs from intact *D. intortum* and a control (empty polyethylene terephthalate bag) for 12 h, starting at the last 2 h of the photophase (Magara et al. 2015; Mutyambai et al. 2015). The aerial parts of the plants were gently enclosed in new sterile PET bags and tied gently around the stem. Precaution was taken not to damage the plants to avoid emission of VOCs in response to mechanical damage. Charcoal-purified air was passed through the inlet port at a flow rate of 600 mL min^−1^. Volatile organic compounds were collected on single-use charcoal filters (0.05 g, 60/80 mesh, Supelco, USA) inserted into the outlet through which air was drawn at 400 mL min^−1^. After trapping, the entrained VOCs were eluted using 250 µL of gas chromatography (GC) grade DCM (Merck, Germany) in 2 mL micro vials (Agilent Technologies, Warsaw, Poland) and stored in a −80 °C freezer before further chemical analysis and bioassays. Entrainments from each host plant were replicated four times and each plant was used only once.

Analysis of VOCs was done using gas chromatography-mass spectrometry (GC–MS) on an HP 7890 A series gas chromatograph (Agilent Technologies, Wilmington, USA) coupled with an HP 5975 C mass spectrometer (Agilent Technologies, Wilmington, USA). The column used was 30 m × 0.25 mm i.d., 0.25 mm Agilent HP-5 MS capillary column. An aliquot (1 µL) of each sample was injected in splitless mode at an oven temperature of 35 °C for 5 min, which gradually increased to 280 °C at 10 °C/min and held for 10.5 min. The carrier gas was helium at a flow rate of 1.0 ml/min and the mass spectra were acquired at 70 eV within a mass range of 38–550 Daltons (Da) during a scan time of 0.73 scans sec-1. Volatiles were identified by comparing their mass spectral data with the GC/MS library data and NIST website (Adams 1995; NIST 2008). Available authentic standards were used to confirm the identities of compounds based on their mass spectral data and retention times (RTs). Quantification of the identified VOCs was done using calibration curves (peak area vs. concentration) generated by serial dilutions of the authentic standards β-pinene and (*E*)-caryophyllene analyzed under the same GC/MS conditions at five different concentrations (1 − 500 ng/mL) since it equally yields results as close to the natural situation as possible. The retention indices (RIs) of the compounds were determined using n-alkane standards (C8-C31).

### Chemicals

Authentic standards of hexanal, (*Z*)−3-hexenol, nonane, α-pinene, octen-3-ol, octanone, (*E*)-β-ocimene, linalool, β-elemene and (*E*)-β-caryophyllene (> 90% purity), all sourced from Sigma Aldrich (Gillingham, UK), were used for quantification and confirmation. Dichloromethane (99.9% purity) used for volatile elution was purchased from Merck (Darmstadt, Germany).

### Coupled Gas Chromatography- Electroantennography

Adult parasitoids of each species were individually collected from the Perspex rearing cage into small cages measuring 40 × 40 × 40 cm. The wasps were subjected to ice at different times to make them immobile. Antennae were prepared by separating the head of ice-chilled wasps from the rest of the body using a scalpel. Two silver-silver chloride (Ag–AgCl) borosilicate glass micro electrodes, 2 mm o.d. X 1.16 mm i.d. with an inner filament (INR-II, Syntech, Hilversum, the Netherlands) were filled with Ringer saline solution as outlined by Maddrell (1969) but without glucose and used for electroantennogram recordings. The Ringer saline solution consisted of 0.7 gl^−1^ NaCl (Loba Chemie, India, ≥ 99.5%), 0.5 gl^−1^ KCl (≥ 99%), 0.2 gl^−1^ CaCl₂ (≥ 99%), 2.5 gl^−1^ MgCl₂ (≥ 99%), 2.72 gl^−1^ KH_2_PO_4_ (≥ 99%) and 9.6 ml KOH (≥ 85%), all purchased from Sigma Aldrich. With the help of an electrode holder, the head was placed at the indifferent electrode with the tip of the antenna touching the recording electrode. The glass tube featured a side hole through which the column effluent was introduced. Volatile Organic Compounds to which the antenna of each wasp responded to were identified on the GC component of the GC-EAG. One µl of the concentrated entrainment sample was injected onto a nonpolar column (HP5-MSI, 30 m × 0.25 mm i.d. × 0.25 μm film thickness), (Agilent Technologies, California, USA) in a HP7890 GC (Agilent Technologies, Palo Alto, USA) equipped with a cool on-column injector and a flame ionization detector (FID). The oven temperature was programmed at 35 °C for 2 min and then programmed at 10 °C min − 1 to 280 °C. Nitrogen was used as the carrier gas. Simultaneous recordings of the EAG and FID responses were obtained with specialized software (Electroantennographic Detection 2015 version 1.2.6, Syntech, Hilversum, The Netherlands). The EAD outlet contained an uninterrupted airflow filtered through charcoal at a rate of 200 mL min − 1 directed to the antenna. A total of six coupled runs were completed for each parasitoid. Only FID peaks corresponding to an EAG peak in at least 3 replicates were considered electro-physiologically active.

### Bioassays with Synthetic Standards

The response of *C. vestalis*, *A. ervi* and *A. colemani* to individual synthetic standards was assessed using Y-tube olfactometer assays. Compounds that elicited a common response based on GC-EAG results were selected for the assays. This included hexanal, (*E*)-β-ocimene and (*E*)-β-caryophyllene. The responses to each compound were evaluated in solution at three concentrations: their putative natural release rates (ng/plant/h), double the natural concentration, and half the natural concentration, as previously described by Fiaboe et al. (2023). The synthetic compounds were prepared by diluting 1 mg/ml of each compound in DCM and then formulating the working solution separately from the stock solution. The different concentrations were then applied to filter paper strips (30 mm diameter) using a micropipette (Drummond Scientific, Broomall, USA), then evaluated against a filter paper strip treated with 10-μL DCM. After evaporation, the treated filter papers were placed in the two glass arms attached at the top of the Y-tube olfactometer. In all tested compounds, 30 gravid female wasps without oviposition exposure were tested.

### Statistical Analysis

Choice data from the Y-tube olfactometer assay were subjected to Chi-Square (χ2) goodness-of-fit test to determine significant differences in the odours choices of the insects (*P* < 0.05). The number of *C*. *vestalis*, *A*. *ervi* and *A*. *colemani* responses to intact plants and synthetic VOCs were converted to percentages using the formula [n/N) × 100], where n is the number of parasitoid responses to a given treatment, while N represents the total number of responses (30). Response values of 1 and 0 were chosen to represent response and non-response, while excluding the non-responsive parasitoid wasps. A Venn diagram was generated to illustrate shared and unique compounds that elicited responses in EAG across the three insects using the Venn Diagram package (version 1.7.3). All data analysis was carried out in R software (v4.3.0) using the R Studio user interface (v2023.06.0) (R Core Team 2023).

## Results

### Behavioral Responses of *C. vestalis*, *A. ervi* and *A. colemani* using Y-tube Olfactometer

**Fig. 1:**
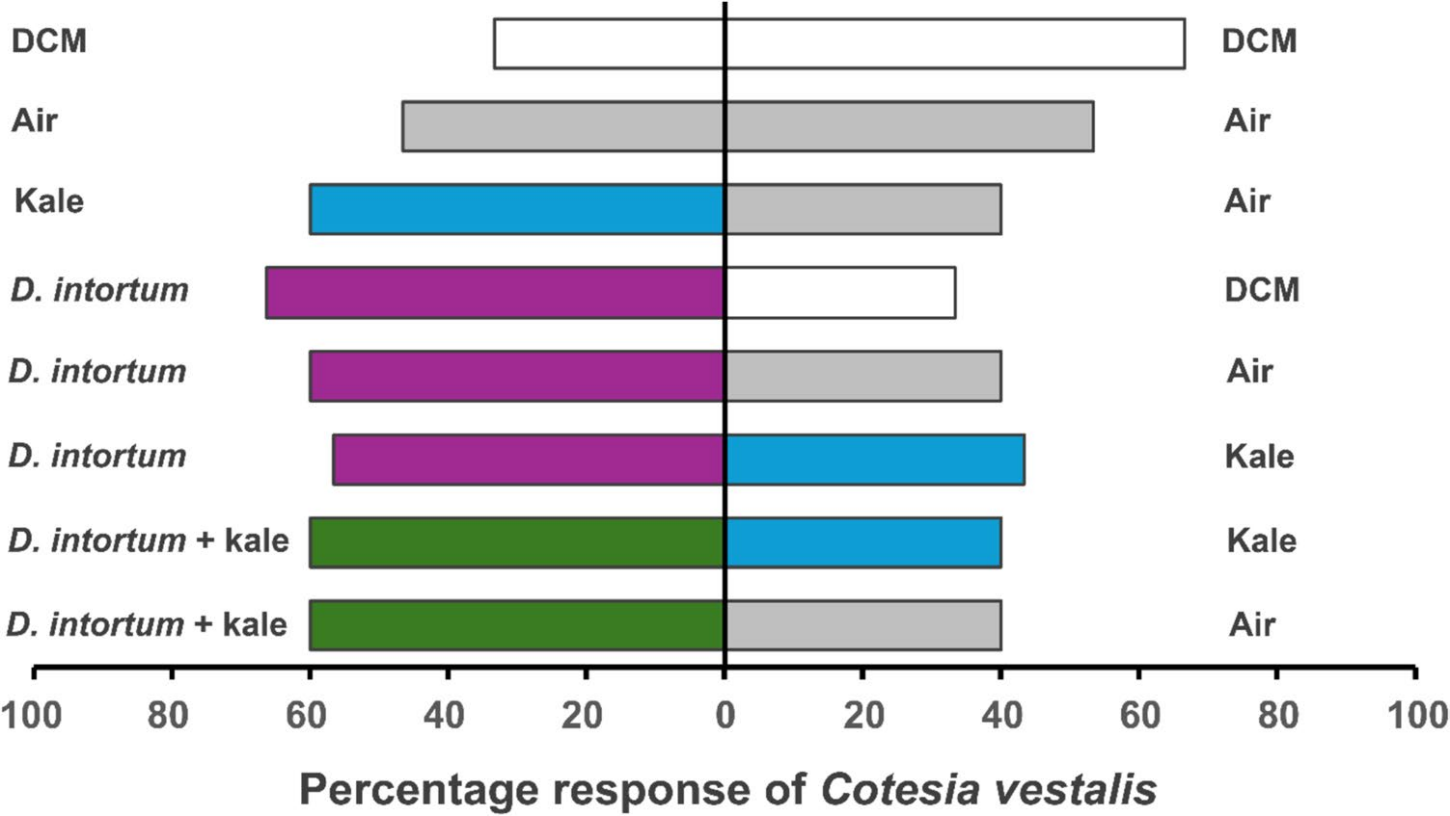
Behavioral responses of *C. vestalis* to volatiles from intact *D. intortum*, kale and control treatments (air and dichloromethane) in a Y-tube olfactometer assay. Bars represent the percentage of choices made by female wasps based on 30 observations. DCM, dichloromethane. Non-responding insects (nr) were excluded from the analysis

Our results show that female *C*. *vestalis* showed no significant response to all the 8 test combinations (*P* > 0.05, Supplementary Table 2). However, *D*. *intortum* elicited more *C*. *vestalis* choices either alone or in combination with kale as compared to sole kale, DCM and empty oven bags. Interestingly, the wasps showed an equal response to the pooled *D*. *intortum* and kale combination compared to kale and empty bag (*X*^2^ = 1.20, df = 1, *P* > 0.05) (Fig. 1, Supplementary Table 2).

**Fig. 2:**
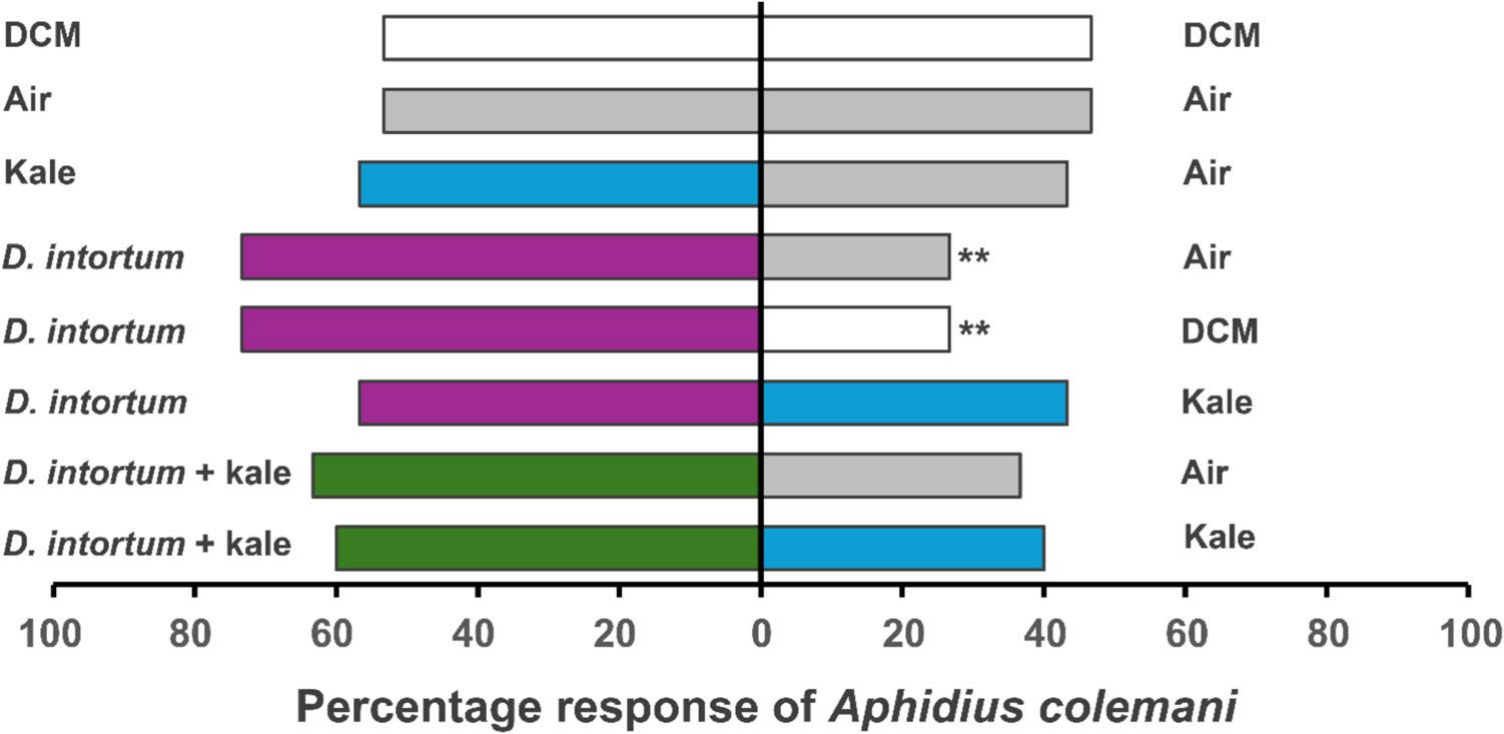
Behavioral responses of *A*. *colemani* to volatiles from intact *D. intortum*, kale and control treatments (air and dichloromethane) in a Y-tube olfactometer assay. Bars represent the percentage of choices made by female wasps based on 30 observations. DCM, dichloromethane. Asterisks (*) indicate significant differences (Chi-square test at α = 0.05). Non-responding insects (nr) were excluded from the analysis

*Aphidius colemani* females exhibited differential responses to the test combinations in the Y-tube olfactometer bioassays. *A*. *colemani* females showed a preference to volatiles of *D*. *intortum* compared to volatiles of DCM (*X*^2^ = 6.53, df = 1, *P* = 0.01) and air (*X*^2^ = 6.53, *P* = 0.01). Despite no significant differences, more choices were recorded in the combination of *D*. *intortum* and kale plants compared to clean air (*X*^2^ = 2.13, df = 1, *P* > 0.05) and sole kale plant (*X*^2^ = 1.20, df = 1, *P* > 0.05), respectively. There were also equal responses when the parasitoid was presented with DCM and clean air in the two arms (*X*^2^ = 0.13, df = 1, *P* > 0.05) (Fig. 2, Supplementary Table 2).

**Fig. 3:**
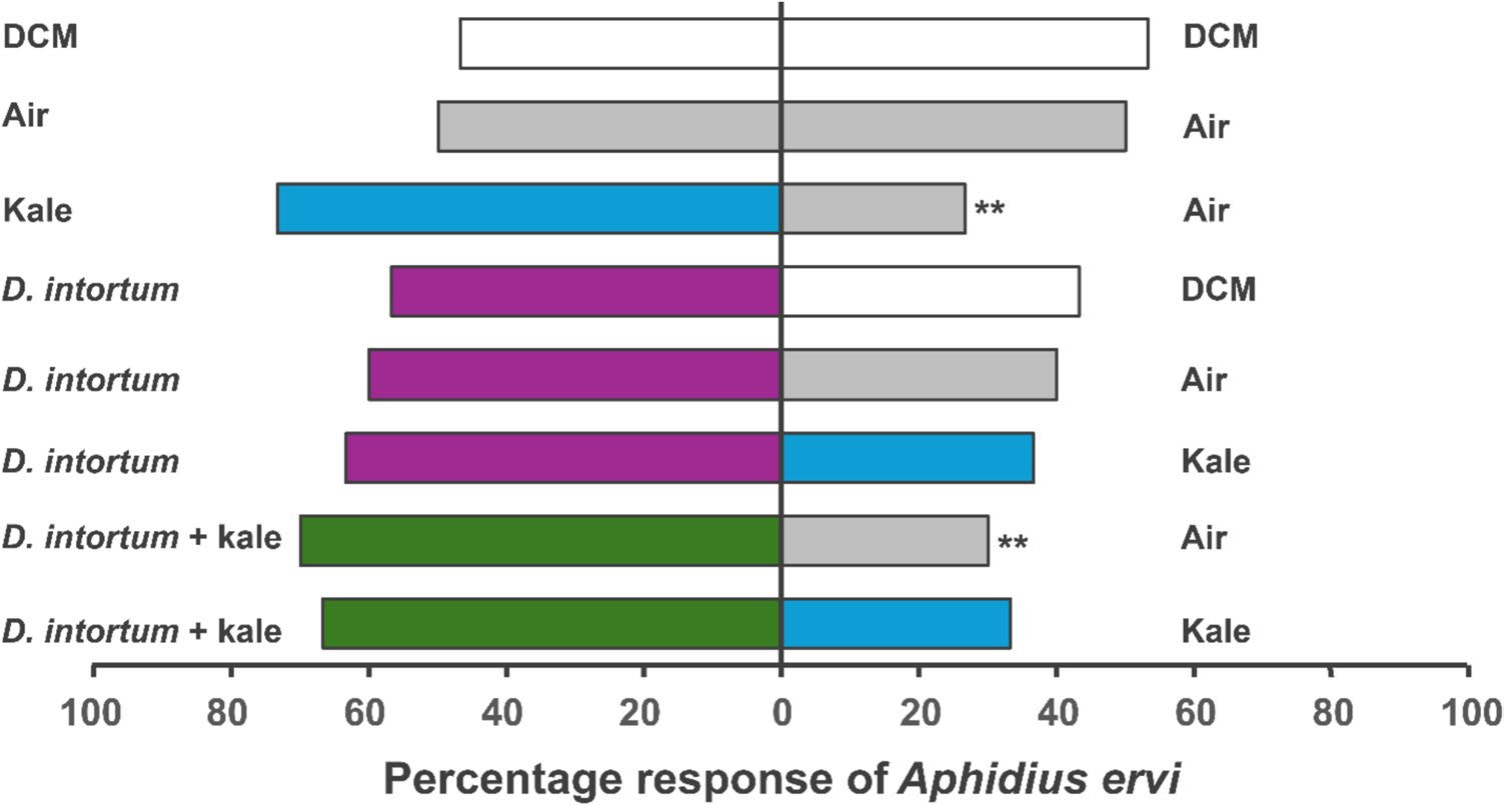
Behavioral responses of *A. ervi* to volatiles from intact *D. intortum*, kale and control treatments (air and dichloromethane) in a Y-tube olfactometer assay. Bars represent the percentage of choices made by female wasps based on 30 observations. DCM, dichloromethane. Asterisks (*) indicate significant differences (Chi-square test at α = 0.05). Non-responding insects (nr) were excluded from the analysis

*Aphidius ervi* females also exhibited varied responses to the test combinations in the Y-tube olfactometer bioassays. The parasitoids were significantly attracted to the combination of *D*. *intortum,* and kale as compared to air (*X*^2^ = 4.80, df = 1, *P* = 0.03) and kale compared to air (*X*^2^ = 6.53, df = 1, *P* = 0.01). Although not significant, the wasps showed more preference to the arms with *D*. *intortum* VOCs as compared to air (*X*^2^ = 1.20, df = 1, *P* > 0.05), DCM (*X*^2^ = 0.53, df = 1, *P* > 0.05) and kale (*X*^2^ = 2.13, df = 1, *P* > 0.05). Similarly, the wasps marginally preferred the arm with the combination of *D*. *intortum* and kale as opposed to the one holding sole kale plant (*X*^2^ = 3.33, df = 1, *P* > 0.05) (Fig. 3, Supplementary Table 2).

### Gas-Chromatography Mass Spectrometry Analysis of *D. intortum* Headspace Volatile Organic Compounds

**Fig. 4:**
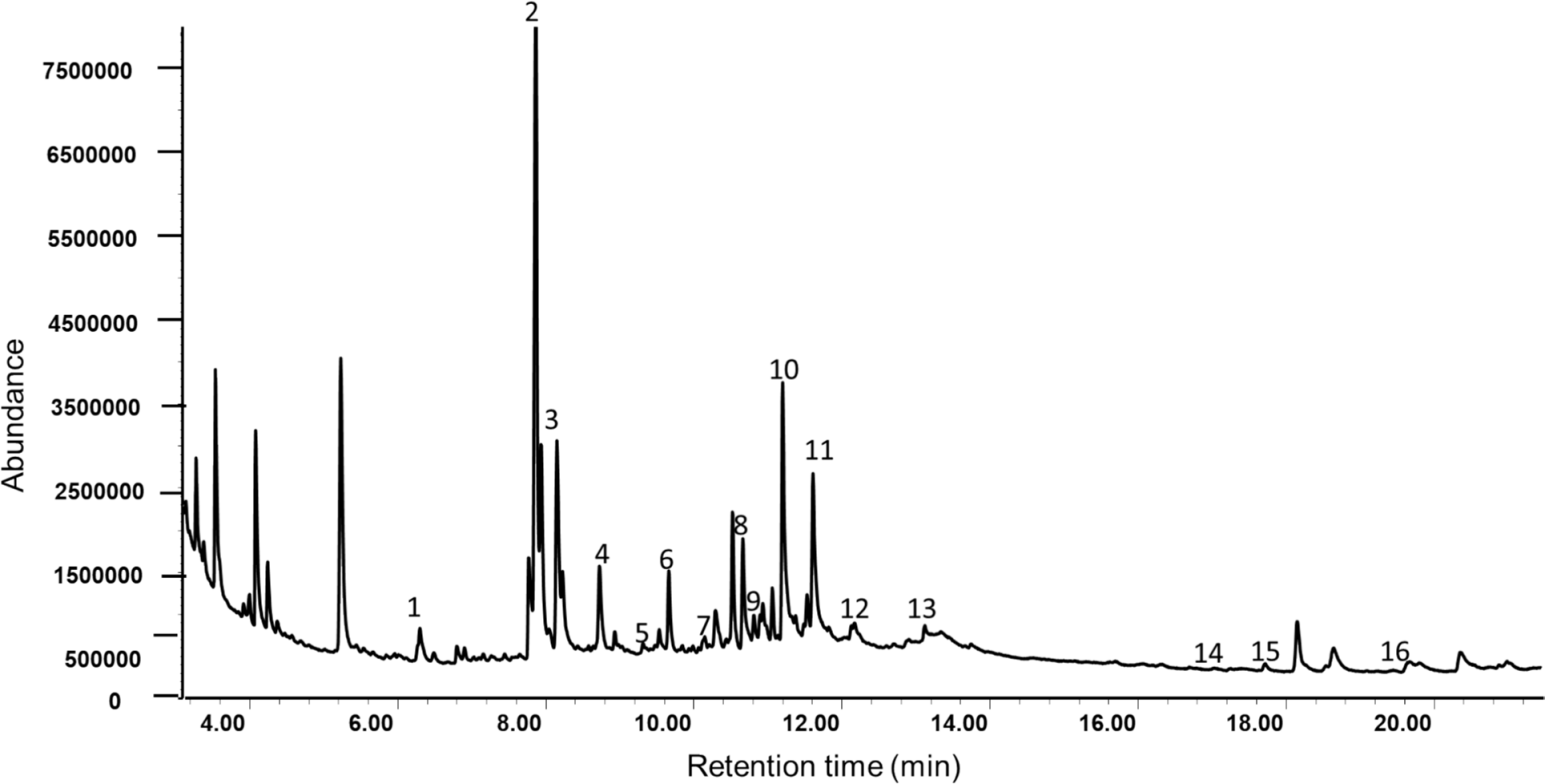
Representative chromatogram of volatile organic compounds identified in *D. intortum* plant headspace volatiles. Identities of the peaks are shown in Table 1

Sixteen different VOCs were detected in the headspace VOCs of *D*. *intortum* (Table 1). Among the VOCs, (*Z*)−3-hexenyl acetate was the most abundant, while nonane was the least abundant VOC (Table 1). Other compounds including α-pinene**,** sabinene, linalool and β-elemene were also identified although at varying mean concentrations (Table 1, Fig. 4).

**Table 1:** Average concentration (ng/plant/h) of volatile organic compounds detected in the headspace samples of D. intortum plant (*n* = 4)

ID	Retention index	Compound	Mean ± SE
1	6.3	Hexanal	153.80 ± 60.37
2	7.8	(*Z*)−3-Hexenol	1390.04 ± 922.17
3	8.1	p-Xylene	601.51 ± 277.72
4	8.7	o-Xylene	315.75 ± 145.38
5	8.9	Nonane	63.07 ± 36.63
6	9.6	α-Pinene	120.88 ± 49.74
7	10.3	Cumene	159.85 ± 98.70
8	10.6	Octen-3-ol	314.78 ± 131.11
9	10.8	Octanone	439.02 ± 200.46
10	11.2	(*Z*)−3-Hexenyl acetate	2676.70 ± 1478.94
11	11.6	Sabinene	400.34 ± 150.76
12	12.0	(*E*)-β-Ocimene	293.35 ± 82.35
13	12.8	Linalool	152.82 ± 66.34
14	17.2	β- Elemene	230.56 ± 79.08
15	17.9	(*E*)-β-Caryophyllene	340.58 ± 124.09
16	19.6	Unknown compound	1393.51 ± 317.81

Values represent averages of independent replicates ± mean standard errors (SE).

### Electrophysiological Responses of *C. vestalis*, *A. colemani* and *A. ervi* to *D. intortum* Volatiles

**Fig. 5:**
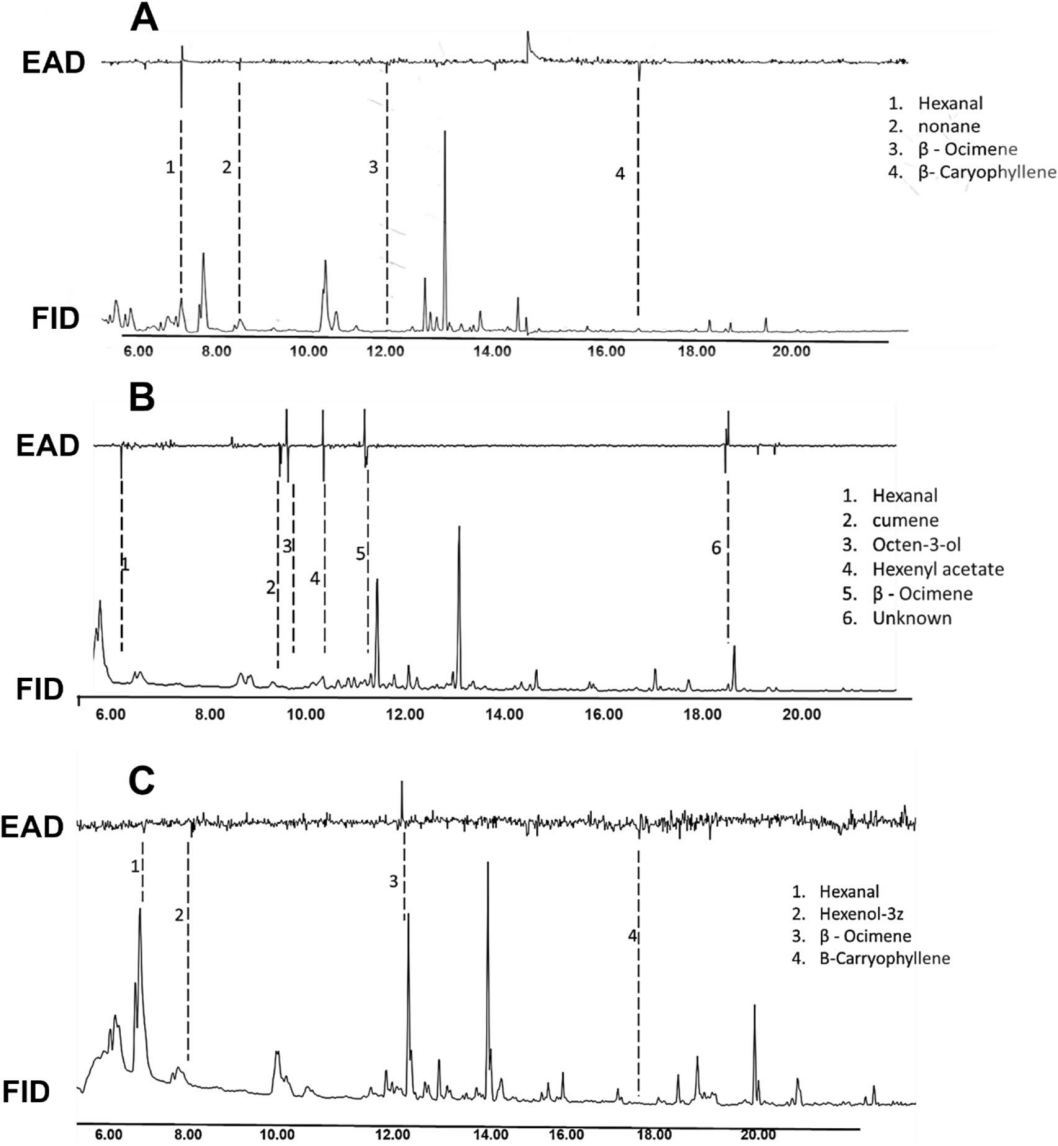
Electrophysiological responses of *C. vestalis* (A), *A*. *colemani* **(B)** and *A. ervi* **(C)** to *D. intortum* headspace volatiles

**Fig. 6:**
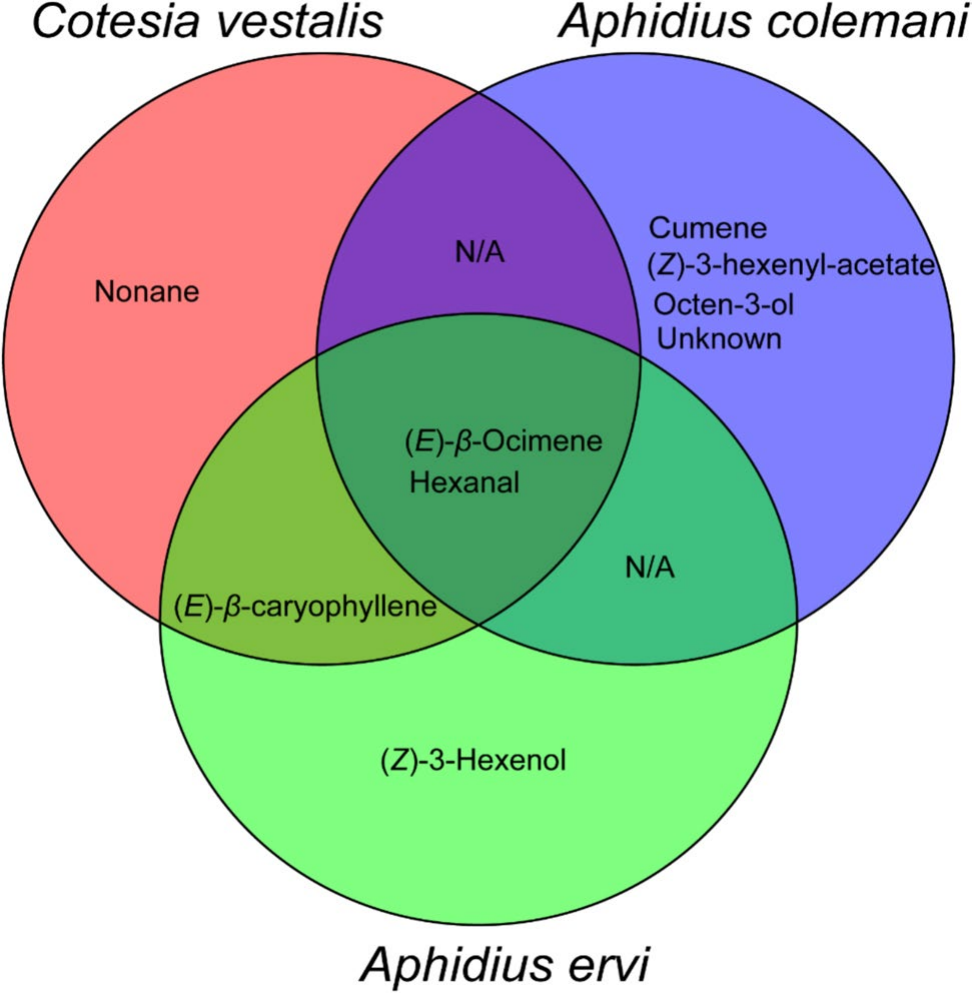
Venn diagram comparing *D. intortum* volatile compounds that elicited electrophysiological responses in *C. vestalis*, *A*. *colemani* and *Aphidius ervi*. N/A indicates no shared compounds

**Fig. 7:**
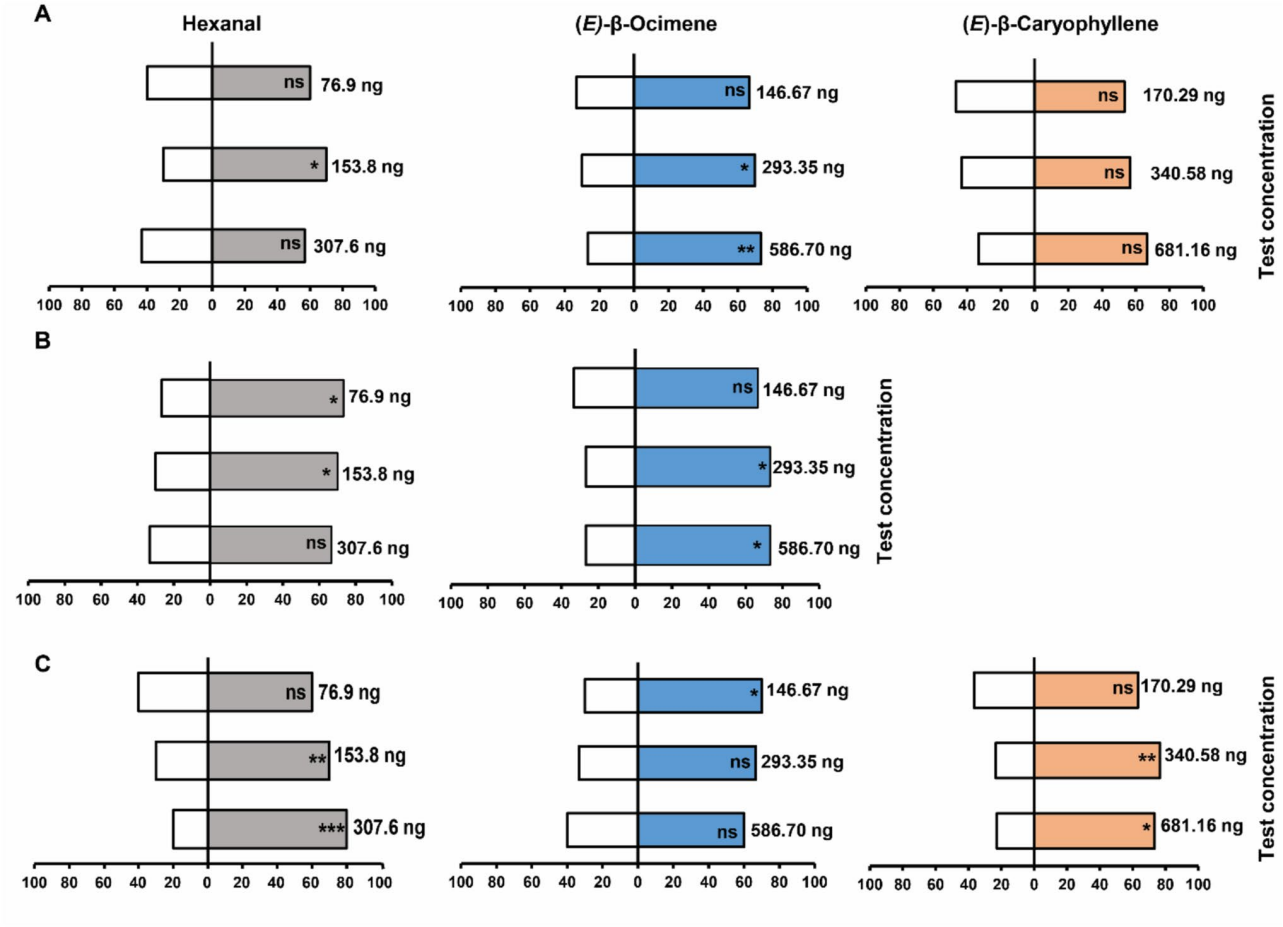
Behavioral responses of *C. vestalis* (A)*, A*. *colemani* (B) *and A. ervi* (C) to synthetic compounds of hexanal, (*E*)-β-ocimene and (*E*)-β-caryophyllene and DCM control in a Y-tube olfactometer assay. Bars represent the percentage of choices made by female wasps based on 30 observations. Asterisks (*) indicate significant differences (Chi square test: *P < 0.05, **P < 0.01, ***P < 0.001)). Non-responding insects (nr) were excluded from the analysis

The antenna of *C. vestalis* detected hexanal, nonane, (*E*)-β-ocimene and (*E*)-β-caryophyllene compounds from *D. intortum*, while that of *A. colemani* detected hexanal, cumene, octen-3-ol, (Z)−3-hexenyl acetate, (*E*)-β-ocimene and an unknown compound (16). Additionally, the antenna of *A. ervi* further detected hexanal, (*Z*)−3-hexenol, β-ocimene and (*E*)-β-caryophyllene. The antennae of all the tested parasitoids commonly detected hexanal and (*E*)-β-ocimene. Both *C. vestalis* and *A. ervi* antennae detected (*E*)-β-caryophyllene. Overall, the antenna of *A. colemani* detected more compounds compared to *C*. *vestalis* and *A*. *ervi* (Fig. 5, 6, 7).

Venn diagram analysis revealed presence of species-specific VOCs across the three insects. The highest number of species-specific VOCs were observed in *A*. *colemani*. These include cumene, octen-3-ol, (*Z*)−3-hexenyl acetate and an unknown compound. On the other hand, (*Z*)−3-hexenol and nonane were the unique VOCs that elicited responses in *A. ervi* and *C. vestalis*, respectively. (*E*)-β-caryophyllene was shared between *C. vestalis* and *A. ervi* while no overlapping VOCs were observed between *C. vestalis* Vs. *A*. *colemani* and *A. ervi* Vs. *A*. *colemani*. Interestingly, (*E*)-β-ocimene and hexanal were commonly shared across the three parasitoids (Fig. 6).

### Behavioral Responses of *A. ervi*, *A. colemani* and *C. vestalis* to Synthetic Compounds

*Cotesia vestalis* was significantly attracted to the natural concentration of hexanal (*X*^2^ = 4.80, df = 1, *P* = 0.03) and avoided the half and double concentrations (*P* > 0.05). Similarly, the plant derived natural concentration (*X*^2^ = 4.80, df = 1, *P* = 0.03) and double concentrations (*X*^2^ = 6.53, df = 1, *P* = 0.01) of (*E*)-β-ocimene significantly attracted the females of *C. vestalis*. In contrast, *C. vestalis* females showed no significant attraction towards all the concentrations of (*E*)-β-caryophyllene, except the double concentration which was marginally attractive (*X*^2^ = 3.33, df = 1, *P* = 0.07) (Fig. 7A**, Supplementary Table 3).** *Aphidius colemani* was significantly attracted to the plant derived (*X*^2^ = 4.80, df = 1, *P* = 0.03) and half (*X*^2^ = 6.53, df = 1, *P* = 0.01) concentrations of hexanal. Conversely, the wasp was significantly attracted to the double (*X*^2^ = 6.53, df = 1, *P* = 0.01) and plant derived natural concentrations of synthetic (*E*)-β-ocimene (*X*^2^ = 6.53, df = 1, *P* = 0.01) (Fig. 7B**, Supplementary Table 3).** On the other hand, *A*. *ervi* exhibited a significant attraction towards the olfactometer arms with double (*X*^2^ = 10.8, df = 1, *P* = 0.001) and plant derived natural concentrations of synthetic hexanal (*X*^2^ = 4.80, df = 1, *P* = 0.02). Similarly, *A. ervi* was significantly attracted to the plant derived natural concentrations (*X*^2^ = 8.53, df = 1, *P* = 0.003) and double (*X*^2^ = 6.53, df = 1, *P* = 0.01) concentrations of (*E*)-β-caryophyllene relative to their solvent controls. In contrast, (*E*)-β-ocimene was only attractive to *A. ervi* at half the putative natural concentration (*X*^2^ = 4.80, df = 1, *P* = 0.03). Nevertheless, the wasp chose more the arms loaded with the natural putative concentration of (*E*)-β-ocimene as compared to the solvent control, although the difference was marginally significant (*P* = 0.07) (Fig. 7C**, Supplementary Table 3).**

## Discussion

The mechanisms of crop protection by *Desmodium* plants are variable with both above and belowground factors reported important in influencing insect behavior (Lang et al., 2023, 2024; Sobhy et al. 2022; Mutyambai et al. 2023) although sometimes contentious (Erdei et al. 2024; Odermatt et al. 2024). Previous field observations from the VIPP system have shown increased protection of kales against aphids and DBM, and *Tuta absoluta* in tomatoes (*Solanum lycopersicum* L.), when intercropped with maize and *Desmodium uncinatum* compared to sole maize with vegetables (Chidawanyika et al. 2025). The reduction in pest damage has been attributed to the overexpression of anti-herbivore metabolites by *D*. *uncinatum*, along with changes in plant–herbivore population dynamics (Chidawanyika et al. 2025). The present study complements these findings by examining how *D. intortum* VOCs modulate the behavior of key kale pest parasitoids including *C. vestalis, A. colemani* and *A. ervi*. Apart from pest repellency, it was hypothesized that the intercropped *D. intortum* within the VIPP recruits parasitoids that may aid defense, as with maize pests and their parasitoids (Sobhy et al. 2022). Our results showed that the two aphid parasitoids, *A*. *colemani* and *A. ervi* were attracted to *D. intortum* VOCs compared to the DBM parasitoid *C. vestalis* (Figs. 1–3). Generally, parasitoids rely on herbivore-induced volatiles (HIPVs) to locate their host, which vary in specific compound ratios, depending on the condition of the plant (Ross et al. 2023). Aphid parasitoids can respond to constitutive plant VOCs since their feeding behaviour causes minor injury, enough to trigger emission of detectable HIPVs (Takemoto & Takabayashi 2015; Ahmed et al. 2022). This indicates that *D. intortum* VOCs likely mimic these HIPV blends, thereby re-directing aphid parasitoids host-searching behavior. Contrary, DBM larval feeding and oviposition can induce significant damage to host plants (Hussain et al. 2020), thereby making constitutive VOCs a lesser determinant of behavioral response. This could explain the less attraction of *C. vestalis* to kale and *D. intortum* treatments. Furthermore, parasitic wasps can locate their host using visual cues, perceived sound or contact stimuli either individually or in concert. This information is processed in the insect sensory system in order to enhance foraging efficiency by reducing the energy and search time (Soyelu 2014). With the processed information, parasitic wasps can improve their host detection by distinguishing plants infested by their hosts from those infested by related but non-host species through behavioural plasticity (Wang et al. 2003). Additionally, the behavioural responses of parasitic wasps varies among individuals of the same species due to their physiological status, experience and their genotype, and therefore the behaviour of generalist species is presumably adaptive (Wang et al. 2003). The geographic isolation of parasitoids and stimuli species can lead to variations in the olfactory receptors due to genetic differences in the target parasitoid or the host plant volatiles (Krishnan et al. 2024).

The GC–MS analyses from our study detected 16 compounds from *D. intortum* (Table 1; Fig. 4 & 5). This is contrary to earlier reports by Erdei et al., (2024) who reported non-emission of bioactive compounds by the plant species. Of these bioactive compounds, EAG recordings from the 3 tested parasitoid showed differential detection (Fig. 6). All the parasitoid species were able to detect (*E*)-β-ocimene and hexanal whilst (*E*)-β-caryophyllene was detected by only *C. vestalis* and *A. ervi*. Additionally, only *C. vestalis* detected nonane whilst *A. colemani* detected cumene, octen-3-ol, (*Z*)−3-hexenyl acetate and an unknown compound. Such differential detection of bioactive compounds by insects is not new and is dependent on several complex factors including sensory structures, diverse receptor proteins, sophisticated neural processing (Barish and Volkan 2015; Zhou and Jander 2022) and even physiological status (Pogue et al. 2024). In our study, all the tested insects were maintained under similar conditions, age and feeding regimes providing similar physiological status. We, therefore, attribute differential antennal responses to the sensory modalities and the subsequent neural processing, which in turn influenced behavioral responses.

Numerous studies have highlighted that parasitic wasps rely on complex olfactory cues and varying ratios of volatile compounds to locate their host (Girling et al. 2011; Kugimiya et al. 2024; Uefune et al. 2020). In a previous study, *C. vestalis* was reported to be attracted to a blend of herbivore-induced plant volatiles (HIPVs) containing α-pinene, sabinene, (Z)−3-hexenyl acetate and n-heptanal in the greenhouse, which were specifically released upon DBM larval feeding (Takemoto & Takabayashi 2015). Additionally, the parasitoid utilizes 1,2-diethyl benzene and 1,4-diethyl benzene, emitted by buckwheat (*Fagopyrum esculentum*) to locate nectar sources, thereby enhancing its survival and efficacy (You et al. 2024). Conversely, *C*. *vestalis* antenna detected nonane in the present study. The lack of preference to *D. intortum* by *C. vestalis* could also be due to lack of herbivore-induced plant volatiles which could elicit behavioural response, an indication that *C. vestalis* relies on specific ratios of blend compositions after active herbivory rather than constitutive emissions. Nonane has been previously associated with evoking *Aphidius gifuensis* responses towards tobacco plants attacked by *Myzus persicae* (Song et al. 2021). On the other hand, *A*. *colemani* exhibits a preference for specific VOCs such as limonene, decane, α-pinene, (*Z*)−3-hexenol identified in intact and aphid-infested Brassicaceous plants (Ahmed et al. 2022). In line with our study, Chehab et al. (2010) reported (*Z*)−3-hexenyl acetate as a signaling VOC of *A*. *colemani*. The unique detection of this VOC may confirm the role of the compound in considerably attracting *A*. *colemani* towards *D. intortum*. *Aphidius ervi* has been reported to be attracted to (*E*)-β-ocimene (Cascone et al. 2023), n-octanal, (*Z*)−3-hexenyl acetate, (*Z*)−3-hexenol, (*E*)-β-caryophyllene and (*E*)-β-farnesene, emitted by infested tomato (*Solanum lycopersicum*) and broad beans (*Vicia faba*) (Takemoto & Takabayashi 2015). (*E*)-β-ocimene has also been reported to stimulate the production of VOCs that confer protection to healthy *S*. *lycopersicum* against aphid infestations and reduce their developmental success (Cascone et al. 2023). Notably, our study also demonstrated that *A*. *ervi* antenna detected hexenal. This VOC has been associated with maize herbivore deterrence and signaling to Braconidae parasitoids. Moreover, some of these VOCs have been identified before as constitutive *D*. *intortum* VOCs in PPT, triggering electrophysiological and behavioral responses in *S*. *frugiperda* and *Cotesia icipe* (Sobhy et al. 2022; Peter et al. 2023). Therefore, it is plausible to attribute pest repellence and natural enemy attraction to the constitutive VOCs emitted by *D*. *intortum***.** It has been established that diversified cropping systems enhance natural enemy populations, boost pest control, and reduce crop damage (Guinet et al. 2023; Sobhy et al. 2022). Our findings offer insights into the plant–parasitoid interactions, further supporting the use of *D*. *intortum* in habitat manipulation against cruciferous pests such as DBMand aphids in VIPP. Such eco-friendly pest management practices enhance food and nutrition security, income, and ensure efficient resource utilization per unit area of available arable lands for resource-poor smallholder farmers (Chidawanyika et al. 2025; Ouya et al. 2023). Additionally, increased production of defensive metabolites reinforces the effect of *D. intortum*, thus contributing to plant–herbivore resistance (Mutyambai et al. 2019). These reported mechanisms could explain why VIPP technology demonstrates high resilience and increased crop productivity (Chidawanyika et al. 2023). Future research should assess the genetic mechanisms underpinning the synthesis and emission of the identified *D*. *intortum* VOCs and their specific role in attracting other vegetable pest parasitoids. This could enhance the precision of pest management within the VIPP framework, thus optimizing the benefits to farmers.

## Conclusion

The present study evaluated the potential of *D. intortum* infochemicals in attracting three key parasitoids of vegetable pests namely *C. vestalis*, *A. ervi*, and *A. colemani*. The aphid parasitoids, *A*. *colemani* and *A*. *ervi* were strongly attracted to *D. intortum* volatiles compared to the DBM parasitoid *C*. *vestalis*. This provides evidence that *D. intortum* volatiles’ appeal to parasitoids is species-specific and not universal. Additionally, EAG recordings from the 3 tested parasitoid demonstrated differential detection, with all the parasitoids responding to (*E*)-β-Ocimene and hexanal. These differential antennal responses could be attributed to the sensory modalities and subsequent neural processing, thus modifying the behavioral responses. Further assays showed that (*E*)-β-ocimene and hexanal have a broad appeal to the tested parasitoids, by eliciting attraction at varying concentrations. However, (*E*)-β-caryophyllene was selectively attractive *A*. *ervi*, with no significant attraction observed in *C*. *vestalis*. Further studies may be required to quantify the recruitment and assemblages of these parasitoids under VIPP field conditions.

## Supplementary Information

Below is the link to the electronic supplementary material.

Supplementary file1 (DOCX 23 KB)

## Data Availability

No datasets were generated or analysed during the current study.
