# Supplementary material for: Testing the Attractive Appeal of Desmodium Infochemicals to Key Parasitoids of the Vegetable Integrated Push–Pull Cropping System
Source: J Chem Ecol. 2025 Jul 4;51(4):73. doi: 10.1007/s10886-025-01622-1 (PMC12226689; doi:10.1007/s10886-025-01622-1)
Supplement: Supplementary file 1 — Supplementary file1 (DOCX 23 KB) [file 10886_2025_1622_MOESM1_ESM.docx]

**Supplementary Table 1.** Pairwise comparison of *C*. *vestalis*, *A*. *ervi* and *A*. *colemani* responses to Kale and Desmodium volatiles

| Experiment | Treatment pairing |
| --- | --- |
| A | *Desmodium* + kale vs. kale |
| B | *Desmodium* + kale vs. Empty bag |
| C | *Desmodium* vs. kale |
| D | *Desmodium* vs. empty bag |
| E | *Desmodium* volatiles vs. DCM |
| F | Kale plant vs. empty bag |
| G | Empty bag vs. empty bag |
| H | DCM vs. DCM |

**Supplementary Table 2**. Results of the Chi-square test for the different treatment combinations

| **Parasitoid species** | **Treatment pairing** | ***X^2^* (df=1)** | ***P* value** |
| --- | --- | --- | --- |
| *Cotesia* *vestalis* | *Desmodium* + kale vs. kale | 1.20 | 0.27 |
|  | *Desmodium* + kale vs. Empty bag | 1.20 | 0.27 |
|  | *Desmodium* vs. kale | 0.53 | 0.47 |
|  | *Desmodium* vs. empty bag | 1.20 | 0.27 |
|  | *Desmodium* volatiles vs. DCM | 3.33 | 0.07 |
|  | Kale plant vs. empty bag | 1.2 | 0.27 |
|  | Empty bag vs. empty bag | 0.13 | 0.72 |
|  | DCM vs. DCM | 3.33 | 0.07 |
| *Aphidius* *ervi* | *Desmodium* + kale vs. kale | 3.33 | 0.07 |
|  | *Desmodium* + kale vs. Empty bag | 4.80 | 0.03 |
|  | *Desmodium* vs. kale | 2.13 | 0.14 |
|  | *Desmodium* vs. empty bag | 1.20 | 0.27 |
|  | *Desmodium* volatiles vs. DCM | 0.53 | 0.47 |
|  | Kale plant vs. empty bag | 6.53 | 0.01 |
|  | Empty bag vs. empty bag | 0.00 | 1.00 |
|  | DCM vs. DCM | 0.13 | 0.71 |
| *Aphidius* *colemani* | *Desmodium* + kale vs. kale | 1.2 | 0.27 |
|  | *Desmodium* + kale vs. Empty bag | 2.13 | 0.14 |
|  | *Desmodium* vs. kale | 5.33 | 0.47 |
|  | *Desmodium* vs. empty bag | 6.53 | 0.01 |
|  | *Desmodium* volatiles vs. DCM | 6.53 | 0.01 |
|  | Kale plant vs. empty bag | 0.53 | 0.47 |
|  | Empty bag vs. empty bag | 0.13 | 0.72 |
|  | DCM vs. DCM | 0.13 | 0.72 |

**Supplementary Table 3**. Results of the Chi-square test for the behavioral responses to different synthetic compound concentrations.

| **Parasitoid species** | **Treatment pairing** | ***X^2^* (df=1)** | ***P* value** |
| --- | --- | --- | --- |
| *Cotesia* *vestalis* | Hexanal half concentration vs. DCM | 1.20 | 0.27 |
|  | Hexanal natural concentration vs. DCM | 4.80 | 0.03 |
|  | Hexanal double concentration vs. DCM | 0.53 | 0.47 |
|  | (*E*)-β-ocimene half concentration vs DCM | 3.33 | 0.07 |
|  | (*E*)-β-ocimene natural concentration vs DCM | 4.80 | 0.03 |
|  | (*E*)-β-ocimene double concentration vs DCM | 6.53 | 0.01 |
|  | (*E*)-β-caryophyllene half concentration vs. DCM | 0.13 | 0.72 |
|  | (*E*)-β-caryophyllene natural concentration vs. DCM | 0.53 | 0.47 |
|  | (*E*)-β-caryophyllene double concentration vs. DCM | 3.33 | 0.07 |
| *Aphidius* *ervi* | Hexanal half concentration vs. DCM | 1.20 | 0.27 |
|  | Hexanal natural concentration vs. DCM | 4.80 | 0.02 |
|  | Hexanal double concentration vs. DCM | 10.80 | 0.001 |
|  | (*E*)-β-ocimene half concentration vs DCM | 4.80 | 0.03 |
|  | (*E*)-β-ocimene natural concentration vs DCM | 3.33 | 0.07 |
|  | (*E*)-β-ocimene double concentration vs DCM | 1.20 | 0.27 |
|  | (*E*)-β-caryophyllene half concentration vs. DCM | 2.13 | 0.14 |
|  | (*E*)-β-caryophyllene natural concentration vs. DCM | 8.53 | 0.003 |
|  | (*E*)-β-caryophyllene double concentration vs. DCM | 6.53 | 0.01 |
| *Aphidius* *colemani* | Hexanal half concentration vs. DCM | 6.53 | 0.01 |
|  | Hexanal natural concentration vs. DCM | 4.80 | 0.03 |
|  | Hexanal double concentration vs. DCM | 3.33 | 0.06 |
|  | (*E*)-β-ocimene half concentration vs DCM | 3.33 | 0.06 |
|  | (*E*)-β-ocimene natural concentration vs DCM | 6.53 | 0.01 |
|  | (*E*)-β-ocimene double concentration vs DCM | 6.53 | 0.01 |
